# Retraction Note: Curcumin synergizes with 5-fluorouracil by impairing AMPK/ULK1-dependent autophagy, AKT activity and enhancing apoptosis in colon cancer cells with tumor growth inhibition in xenograft mice

**DOI:** 10.1186/s13046-022-02409-y

**Published:** 2022-06-10

**Authors:** Pan Zhang, Ze-Lin Lai, Hui-Fen Chen, Min Zhang, An Wang, Tao Jia, Wen-Qin Sun, Xi-Min Zhu, Xiao-Feng Chen, Zheng Zhao, Jun Zhang

**Affiliations:** 1 Key Laboratory of Brain Functional Genomics (East China Normal University), Ministry of Education, School of Life Sciences, East China Normal University, Shanghai, 200062 China; 2 Department of Clinical Laboratory, Shanghai First Maternity and Infant Hospital, Tongji University School of Medicine, Shanghai, 201204 China; 3 Department of Clinical Laboratory, Shanghai Public Health Clinical Center Affiliated to Fudan University, Shanghai, 201508 China; 4 Department of Thoracic Surgery, Huashan Hospital, Fudan University, Shanghai, 200040 China; 5 INSERM-UGA U1209, CNRS UMR5309, Institute for Advanced Biosciences, F-38700 La Tronche, France

**Retraction Note: J Exp Clin Cancer Res 36, 190 (2017)**

**https://doi.org/10.1186/s13046-017-0661-7**

The Editor-in-Chief is retracting this article. Concerns have been raised about the welfare of the mice used in this study. The Editor-in-Chief has not been able to obtain clarification on whether the tumour volume and the humane endpoints in the experiments described were approved by the institutional animal ethics committee. None of the authors has responded to correspondence from the Publisher about this retraction.

